# Neglecting Rice Milling Yield and Quality Underestimates Economic Losses from High-Temperature Stress

**DOI:** 10.1371/journal.pone.0072157

**Published:** 2013-08-22

**Authors:** Nathaniel B. Lyman, Krishna S. V. Jagadish, L. Lanier Nalley, Bruce L. Dixon, Terry Siebenmorgen

**Affiliations:** 1 Department of Agricultural Economics and Agribusiness, University of Arkansas, Fayetteville, Arkansas, United States of America; 2 Crop and Environmental Sciences Division, International Rice Research Institute, Metro Manila, Philippines; 3 Department of Food Science, University of Arkansas, Fayetteville, Arkansas, United States of America; Kansas State University, United States of America

## Abstract

Future increases in global surface temperature threaten those worldwide who depend on rice production for their livelihoods and food security. Past analyses of high-temperature stress on rice production have focused on paddy yield and have failed to account for the detrimental impact of high temperatures on milling quality outcomes, which ultimately determine edible (marketable) rice yield and market value. Using genotype specific rice yield and milling quality data on six common rice varieties from Arkansas, USA, combined with on-site, half-hourly and daily temperature observations, we show a nonlinear effect of high-temperature stress exposure on yield and milling quality. A 1°C increase in average growing season temperature reduces paddy yield by 6.2%, total milled rice yield by 7.1% to 8.0%, head rice yield by 9.0% to 13.8%, and total milling revenue by 8.1% to 11.0%, across genotypes. Our results indicate that failure to account for changes in milling quality leads to understatement of the impacts of high temperatures on rice production outcomes. These dramatic losses result from reduced paddy yield and increased percentages of chalky and broken kernels, which together decrease the quantity and market value of milled rice. Recently published estimates show paddy yield reductions of up to 10% across the major rice-producing regions of South and Southeast Asia due to rising temperatures. The results of our study suggest that the often-cited 10% figure underestimates the economic implications of climate change for rice producers, thus potentially threatening future food security for global rice producers and consumers.

## Introduction

Rice (*Oryza sativa* L.) production serves as the primary source of income for nearly one billion people and provides the staple food for more than half the world’s population [1], which is projected to grow from seven to nine billion by 2050 and to reach ten billion before 2100 [2]. Global demand for rice is expected to increase roughly 35% by 2035 [3] – outpacing population growth – requiring an additional 116 million tons of milled rice [4]. Meeting the increasing demand for rice will require improvements in rice production, which is becoming increasingly challenging given the pace at which the climate is changing [5].

Current Intergovernmental Panel on Climate Change (IPCC) models project mean global surface temperature increases between 1.8°C and 4°C over the 21st century at a rate of 0.2°C increase per decade [6]. As surface temperatures increase gradually, the likelihood of both the intensity and the magnitude of high-temperature events could increase substantially [7], thus reducing agricultural crop yields [8], [9], including rice [10]. Recent analyses of climate change’s effects on rice production have estimated the impact of high temperatures on paddy (rough) rice yield [10], [11] and milling quality outcomes, which ultimately determine edible rice yield[12]–[17]. However, the estimation of high temperature effects on yield or milling quality independently of the other outcomes inevitably leads to an underestimation of food security and economic implications because yield and milling quality jointly determine edible rice yield. In spite of the exhaustive literature documented on the negative impact of high-temperature stress on rice yields, there is a void in the literature on the overall market revenue losses associated with increasing temperatures.

Rice yield and milling quality jointly determine the economic value of rice from the field to the mill and in the market. Production shocks in any given stage affect value creation in later stages. Producer revenue depends on the sale of paddy (unprocessed) rice and miller revenue depends on the sale of milled rice and byproducts to domestic and international markets. High-temperature stress can decrease the quantity of paddy (rough, unprocessed) rice available for milling and change the distribution and quality of milling outcomes. Milling quality aspects affected by temperature include chalkiness, immature kernels, kernel dimensions, fissuring, protein content, amylose content and amylopectin chain length [5]. The milled outcomes most important to global human nutrition are the quantities of whole and broken kernels that remain after hulling and milling paddy rice. High temperatures have been shown to decrease the quantity of whole kernel head rice and increase the quantity of broken kernels [12], [14], [17], which sell for approximately 60% of the value of head rice in the U.S. and often for less in Asian markets [18]. Chalkiness – an opaque white discoloration of the endosperm – reduces the value of head rice kernels and decreases the ratio of head to broken rice produced during the milling process [19].

**Figure 1:**
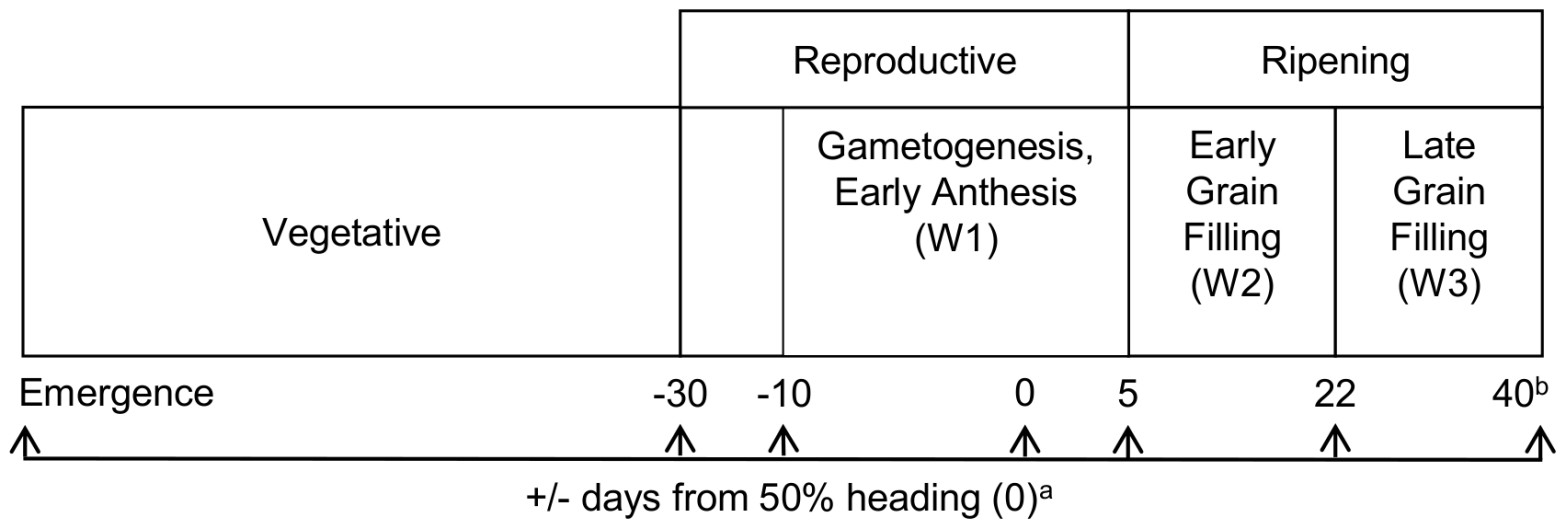
Rice (*Oryza sativa* L.) developmental stages. This figure describes the developmental stages relevant to this study. **a,** Vegetative, reproductive and ripening growth stages are defined relative to the observed 50% heading and emergence dates at each station. The vegetative, reproductive, and ripening stages are defined as the intervals [emergence, H –30 ), [H –30, H +5 ], (H +5, harvest ], respectively, where H denotes 50% heading. The ripening stage is divided into early and late grain filling denoted by W2 and W3 to account for the differential effects of temperature on the physiological processes occurring during these periods. **b,** Harvest dates are not available for the paddy yield data, so we approximate harvest as 40 days after 50% heading. Harvest dates are available for the milled rice yield and quality data, but some plots were not harvested at maturity to allow harvest moisture content (*HMC*) to decrease. To avoid inclusion of temperatures beyond maturity, H +40, harvest is used as the harvest date for milling yield and quality trials.

Recent research has shown that modest (1°C) increases in daily maximum and minimum temperatures can decrease paddy yields by as much as 10% [10], [11], dramatically alter the distribution of head and broken rice [16], [17], and greatly increase the proportion of chalky kernels [15], [16], [17], [19]. Despite the importance of milling outcomes to global food security and the economy, the research to date on paddy yield and milling quality does not intersect, leaving a significant gap in the literature on a crucial topic. We attempt to fill this gap by modeling paddy yield and milling quality as temporal functions of cumulative exposure to day temperatures above 33°C and night temperatures above 22°C during sensitive growth windows (Fig. 1). We estimate high temperature effects on paddy yield – the mass (kg ha^−1^) of unprocessed kernels; milled rice yield (*MRY*) – the mass percentage of milled kernels (milled rice) to an initial mass of paddy rice kernels; head rice yield (*HRY*) – the mass percentage of milled kernels ≥ three-quarters the length of an unbroken (whole) milled kernel (head rice) to an initial mass of paddy rice; broken rice yield (*BKY*) – the mass percentage of milled kernels<three-quarters the length of an unbroken (whole) milled kernel to an initial mass of paddy rice; and chalk content (*CHK*) – defined as the ratio of chalky to non-chalky area of 100 brown rice (kernels obtained immediately after removing the husk) kernels. Definitions of *MRY*, *HRY*, *BKY*, and *CHK* correspond to the experimental definitions stated in [16]. Estimating these components allows predictions of reductions in yield and milling quality attributable to growth-stage-specific, diurnal high-temperature events. We use these estimates to predict changes in paddy yield, chalk, *HRY*, and *MRY* given 1°C, 2°C, and 4°C increases in growing season air temperature. The predictions are used to calculate changes in the quantity and value of paddy and milled rice for producers and millers across temperature scenarios.

## Materials and Methods

### Experimental Data Collection and Definitions

Annual Arkansas Rice Performance Trial (ARPT) experimental data from six locations over four years were used to estimate the yield and milling quality in the models (Table 1). Three randomized plots of each cultivar were planted in each location-year combination and cultivated under standardized management practices for obtaining optimal yields and milling quality [16]. In 2007, 2008, and 2009, the cultivars were harvested over a range of harvest moisture contents (*HMC*) and milled in duplicate. *HMC* is an in-field measurement of the ratio of liquid to solid kernel mass and is expressed as a percentage. *HMC* was measured immediately after samples were harvested using a single-kernel moisture meter (Shizuoka Seiki CTR 800E, Shizuoka, Japan); moisture content was expressed on a wet basis [16]. In 2010, the cultivars were harvested at targeted moisture contents based on optimal harvest moisture content [20] and milled in duplicate. The change in harvest procedure resulted in fewer observations for each cultivar in 2010 than in 2007, 2008 and 2009. In each year, *HMC* and *CHK* were recorded for each harvest repetition and *MRY* and *HRY* were recorded for each milling repetition. Only the *MRY* and *HRY* observations associated with the measured chalk content were used in the present analysis.

**Table 1:** Sample means (standard deviations) and observations (N†) by station.

		Yield	*CHK*	*MRY*	*HRY*	Average Temp. (°C)			
Location	Latitude/longitude	kg ha^−1^	%	%	%	Veg.	W1	W2	W3
Corning	36.4°N/90.6°W	10,551	5.1	71.9	61.7	24.4	25.8	27.0	26.5
N = 34/133		(1,706)	(3.7)	(1.8)	(8.0)	(0.6)	(0.9)	(2.0)	(2.3)
Keiser	35.7°N/90.1°W	8,972	3.6	66.8	55.7	27.0	26.0	25.7	20.3
N = 29/36		(1,296)	(2.9)	(4.6)	(7.8)	(0.6)	(3.4)	(2.6)	(4.1)
Newport	35.6°N/91.3°W	10,334	3.8	71.0	64.4	26.2	27.6	25.9	25.5
N = 43/75		(3,070)	(2.2)	(1.7)	(5.2)	(2.6)	(2.0)	(1.5)	(2.8)
Pine Tree	35.1°N/90.9°W	8,454	3.0	72.3	67.0	25.7	27.4	25.5	23.8
N = 44/93		(1,161)	(1.8)	(2.3)	(5.7)	(1.1)	(1.7)	(3.1)	(1.7)
Rohwer	33.8°N/91.3°W	7,914	6.0	69.8	60.8	25.5	27.3	26.7	26.3
N = 62/157		(1,230)	(4.7)	(3.0)	(10.2)	(0.5)	(1.1)	(2.7)	(2.5)
Stuttgart	34.5°N/91.4°W	9,211	3.8	70.4	63.6	25.1	27.1	27.8	25.9
N = 76/215		(1,272)	(2.5)	(2.2)	(5.9)	(0.5)	(1.4)	(2.1)	(2.1)

This table shows the locations of experiment stations in Arkansas, USA, and the total number of observations (N = paddy/milling) over the study period of 2007–10.

† Paddy yield/milling quality observations were obtained from separate studies carried out in the same locations. Milled observations were obtained in triplicate from samples harvested three times during the season; thus, more milling observations were available for our study.

*MRY* is calculated as the mass percentage of polished whole and broken kernels remaining after milling a 150-gram sample of paddy rice:
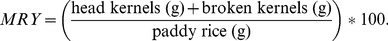
(1)


*HRY* is calculated as the mass percentage of polished whole kernels remaining after milling a 150 gram sample of paddy rice and separation of broken kernels using a double-tray sizing device (Seedburo Equipment Co., Chicago, IL):
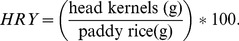
(2)


Broken rice yield (*BKY*) is calculated as the difference between the observed *MRY* and *HRY* because this difference has not been measured experimentally, but is by definition the difference between the two values. Chalk content represents the percentage chalky area of a 100 kernel sample of brown rice, obtained by [16] using a WinSeedle Pro 2005a™ (Reagent Instruments Inc., Sainte-Foy, Quebec, Canada):
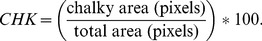
(3)


Ambient air temperature and relative humidity recordings were collected at each location at 30-minute intervals using two temperature sensors (HOBO Pro/Temperature Data Logger, Onset Computer Co., Bourne, MA). The sensors were placed at panicle height, in-between experimental plots, within the 18-plot block of rice cultivar plots grown at each station. Given the randomized block design of cultivar location within each field, this study uses the set of means of each pair of 30-minute temperature observations as the set of temperatures associated with a given year-location combination. We define the thermal time metrics using the half-hourly data points. Thermal days (TD) above 33°C are defined as.
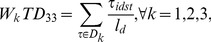
(4)where *W_k_* is window *k* (1,2,3), 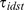 is the temperature at time *i* on day *d* at station *s* in year *t*, 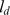
is the length of day *d* and 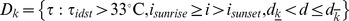, where 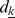 and 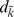 represent the start and end day of window *k*, respectively. Similarly, thermal nights (TN) above 22°C are defined as
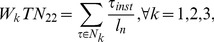
(5)where 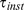 is the temperature at time *i* on night *n* at station *s* in year *t*, 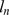 is the length of night *n* and, 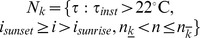, where 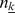 and 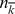 represent the start and end night of window k, respectively.

Daily sunrise and sunset estimates were calculated for each station/year combination using the National Oceanic and Atmospheric Administration’s (NOAA) solar calculator (www.srrb.noaa.gov/highlights/sunrise/sunrise.html). Vapor pressure deficit (*VPD*) (kPa) was calculated using these data following [21]:
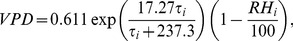
(6)where *RH_i_* is the relative humidity at time *i*.

Half-hourly weather data were not available until 10 days prior to 50 percent heading (i.e. an extension to the vegetative stage which included panicle initiation and early development of panicles; illustrated in Fig. 1) at any experiment station because initially research objectives were concerned mainly with high temperatures during the grain-filling period. Therefore, nearby weather station data was used for temperature data during the early reproductive and vegetative growth stages. Daily mean minimum and maximum temperatures (°C) from nearby weather stations were used to measure temperatures during the early reproductive and vegetative growth stages. These data were obtained from the National Oceanic and Atmospheric Administration weather stations nearest each experiment station [22]. Daily, averaged insolation on horizontal surface (mJ m^−2^) data for 2007–2010 were obtained from the the NASA Climatology Resource for Agroclimatology [23].

### Paddy Yield Model

Paddy yield was estimated using a two-way fixed-effects OLS multiple regression model of the form
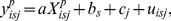
(7)where 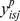 is the natural logarithm of paddy yield (kg ha^−1^) for trial *i* at station *s* for cultivar *j*; 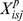 is a vector of weather variables for that trial; *a* is a vector of weather coefficients; *b_s_* is a vector of station intercepts to control for spatially invariant unobserved effects such as soil type; *c_j_* is a vector of cultivar intercepts to capture genetic yield differences across cultivars; and *u_isj_* are the error terms associated with each trial. Heteroskedasticity robust standard errors are used to accommodate non-constant variance across locations and years [24]. The weather variables in 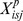 include: 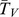, average daily temperature (°C) from emergence to 10 days prior to 50% heading; 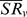, mean daily average solar radiation (mJ m^−2^ d^−1^) during the vegetative stage; 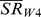, mean daily average solar radiation over the period of 10 days before 50% heading to harvest; and *TDN_k_*, the sum of thermal degree-days above 33°C *TD_33_* and thermal degree-nights above 22°C *TN_22_* during window *k* for *k* = W1, W2, and W3 (Fig. 1).

In-field, half-hourly temperature observations during sensitive growth stages with *TD_33_* and *TN_22_* allow cumulative measurements of exposure to temperatures above day and night thresholds shown harmful to sensitive developmental processes such as pollen development and anthesis, resulting in increased spikelet sterility [25], [26] (Fig. S1). The analysis includes *TDN* = *TD_33_*+ *TN_22_* instead of *TD_33_* and *TN_22_* separately because these variables are highly correlated and the resulting multicollinearity can prevent meaningful interpretation of individual parameters [27]. The combined variable allowed inclusion of stage-specific weather variables instead of aggregating temperature across an entire season, a phenomenon highlighted in [28], where overall rice yield reductions were previously attributed to just one weather variable by [11]. This problem is encountered in weather data with highly correlated day and night temperatures [29]. Nonetheless, *TDN* captures cumulative exposure to high temperatures similar to those used in previous studies [8], [30].

### Milling Quality Model

We estimate a system of linear, fixed-effects equations to capture the dynamic relationship between *CHK*, *HRY*, and *MRY*:
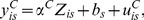
(8)

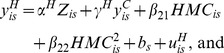
(9)

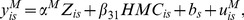
(10)where 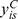, 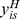, and 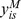
denote chalk content (%), *HRY* (%), and *MRY* (%), respectively, for trial *i* at station *s*; 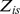
denotes the vector of the same weather variables included in equations (8), (9), and (10); 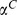, 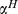, and 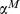
are the vectors of coefficients associated with the weather variables in 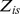; 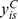 appears on the right hand side (RHS) of equation (9) as an endogenous explanatory variable; 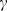 are the slope parameters for 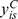 in (9); *HMC_is_* represents harvest moisture content (*HMC*) in equations (9) and (10); 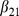,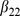, and 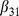 are the slope parameters associated with *HMC* and *HMC^2^* in equations (9) and (10); *b_s_* is a vector of station intercepts; and, 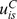, 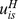, and 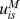
are error terms for each observation. The *HMC^2^* term allows for a quadratic response of *HRY* to *HMC*, a relationship previously documented for these cultivars [20]. Weather variables in 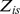 include *TDN_W2_* and *TDN_W3_* to capture temperature effects during early (W2) and late (W3) grain filling (Fig. 1). *HMC* is included on the RHS of equations (9) and (10) to control for reductions in *HRY* due to fissured and immature kernels [20] and to disentangle reductions in *HRY* and *MRY* attributable to high-temperature exposure due to early or late harvest. We estimate both direct and indirect effects of high temperatures on *HRY* by including 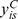 and 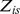 on the RHS of equation (9), calculating the vector of indirect effects of high temperatures on *HRY* as 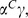
the product of the effect of high temperatures on chalk and the effect of chalk on *HRY*. The vector of direct effects of high-temperature stress is 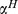, thus the vector of total effects of high temperature exposure on *HRY* is calculated as 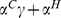.

Estimation of the system by Generalized Method of Moments (GMM) accounts for the heteroskedasticity of unknown forms and allows calculation and statistical testing of both direct and indirect effects. Given the presence of 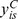 as an explanatory variable in equation (9), we tested and did not reject the null hypothesis of no correlation between 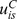 and 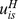. Rejection would have required using instrumental variables in the GMM estimation.

Changes in milling quality given 1°C, 2°C, and 4°C increases in growing season (emergence to harvest) temperatures are estimated using the total effects measure 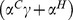. Expected changes in the conditional means of *CHK*, *HRY* and *MRY* are estimated given 1°C, 2°C, and 4°C increases in mean growing season temperature. Using the means of *TDN_W2_* and *TDN_W3_* as the baseline, we then attempt to answer the question: How might the observed sample means of chalk content, *HRY*, and *MRY* change if the data were resampled under the three warming scenarios? To estimate these changes, we add 1°C, 2°C and 4°C to each observed temperature datum following the procedure used in [8], [9]. *TDN_W2_* and *TDN_W3_* are then recalculated for each hypothetical scenario. Then, the sample means of these hypothetical variables are calculated and used to predict changes in the means of the outcome variables under each scenario. Estimating changes in the sample mean of the temperature variables under these scenarios rather than the prediction of each individual observation across scenarios avoids out-of-sample prediction. Investigation of the changes in the sample means under these scenarios rather than any one observation better fits this examination of the future of Arkansas rice production given a warmer climate.

## Results

### Paddy Yield

**Figure 2:**
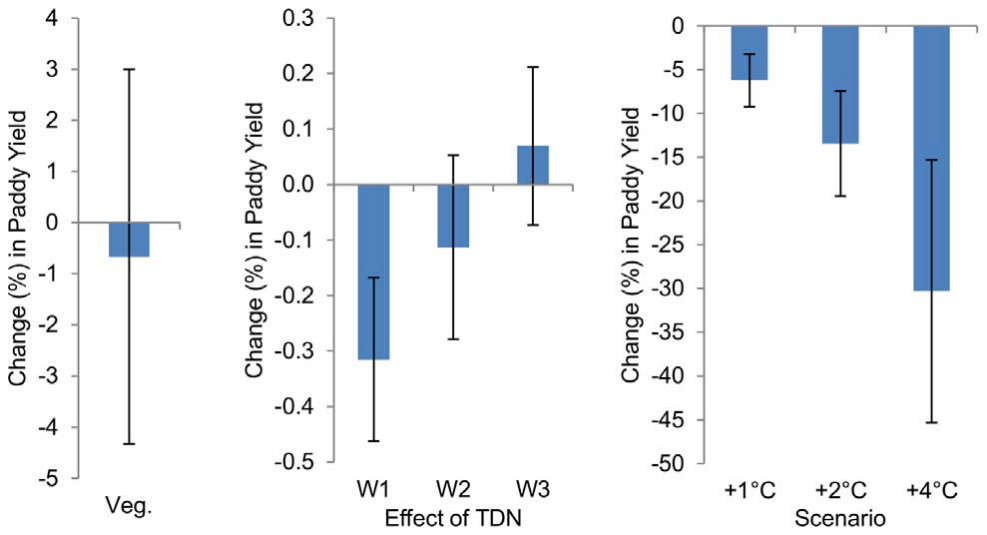
High temperature effects on paddy rice yield. Represented above are the marginal effects and total effects of high temperature on paddy yield. **a**, Marginal effect of a 1°C increase in average daily temperature during the vegetative stage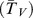 on paddy yield. **b**, Marginal effects of an additional unit of high temperature exposure, *TDN*, during W1, W2, and W3. **c**, Response of mean paddy yield to 1°C, 2°C, and 4°C increases in average growing season temperature. Error bars indicate 95% confidence intervals calculated using heteroskedasticity robust standard errors.

Paddy yield estimates provide a baseline from which we examine the compounding effects of high-temperature stress on milling quality. The estimated effect of average daily temperature during the vegetative stage 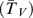 in the paddy model is negative, but statistically insignificant (P>0.10) (Fig. 2a). The effect of high-temperature stress during gametogenesis and early anthesis (W1) is negative and statistically significant (P<0.01), suggesting that a one-unit change in *TDN_W1_* is associated with a -0.3 percent change in paddy yield, all else held constant (Fig. 2b). The *TDN_W2_* and *TDN_W3_* coefficients are statistically insignificant (P>0.10). In paddy yield models with vapor pressure deficit (*VPD*) as an independent variable, the *VPD* coefficients were statistically insignificant and/or unstable across alternate specifications (Table S1, specifications 2 and 3 in File S1).

A 1°C increase in average growing-season temperature implies a 6.2% decline in paddy yield, all else held constant (Fig. 2c). Given 2°C and 4°C increases in growing-season temperature, yield is predicted to decline by 13.5% and 30%, respectively, all else held constant. All three predicted changes in yield are statistically significant (P<0.01) (Fig. 2c).

### Milling Quality

Estimation of the milling quality model yields statistically significant, detrimental effects of high-temperature stress on all elements of milling quality with significant variation across included genotypes (Table 2). In the chalk model (Table 2a), the coefficients on *TDN_W2_* and *TDN_W3_* are positive and statistically significant (P<0.05 to P<0.01) across all cultivars. The magnitudes of the *TDN_W3_* coefficients are larger, suggesting that high-temperature stress during late grain filling has a greater impact on chalk formation. In the *HRY* and *MRY* equations, *TDN_W2_* has a negative, statistically significant coefficient across all cultivars (P<0.10 to P<0.01) and the *TDN_W3_* coefficients are relatively small in magnitude and statistical significance (Table 2b,c).

**Table 2:** Milling yield and quality system estimates.

a. *CHK*	Bengal	Jupiter	Cypress	LaGrue	Wells	XL723
*TDN_W2_*	0.007**	0.014***	0.022***	0.063***	0.043***	0.068***
*TDN_W3_*	0.015***	0.057***	0.039***	0.096***	0.081***	0.105***
Adj. R^2^	0.41	0.77	0.62	0.79	0.68	0.77
**b. ** ***HRY***	**Bengal**	**Jupiter**	**Cypress**	**LaGrue**	**Wells**	**XL723**
*TDN_W2_*	−0.096***	−0.043*	−0.137***	−0.155***	−0.101***	−0.133***
*TDN_W3_*	−0.039*	−0.025	0.079**	0.041	0.020	−0.048*
*CHK*	−0.045	−1.177**	−0.314	−1.140***	−1.859***	−0.478**
*HMC*	2.304**	1.544**	−0.247	−0.706	4.531***	0.975
*HMC^2^*	−0.050**	−0.036**	0.006	0.012	−0.107***	−0.029
Adj. R^2^	0.45	0.45	0.52	0.78	0.79	0.69
**c. ** ***MRY***	**Bengal**	**Jupiter**	**Cypress**	**LaGrue**	**Wells**	**XL723**
*TDN_W2_*	−0.039***	−0.052***	−0.054***	−0.062***	−0.049***	−0.068***
*TDN_W3_*	−0.008	−0.004	0.014	−0.007	−0.021***	−0.021**
*HMC*	−0.129***	−0.270***	−0.066*	−0.191***	−0.161***	−0.210***
Adj. R^2^	0.59	0.72	0.61	0.69	0.59	0.55

**a**, Marginal effects of *TDN* on chalk content (*CHK*) formation; this effect is interpreted as the percentage point change in *CHK* due to a one-unit change in TDN. **b**, Marginal effects of TDN, CHK, and harvest moisture content (*HMC*) on *HRY*. **c,** Marginal effects of *TDN* and *HMC* on M*R*Y. *,**, and *** represent statistical significance at the 0.10, 0.05, and 0.01 levels, respectively. Station fixed-effect estimates have been omitted from this table. We estimated the system using Generalized Method of Moments (GMM) estimation to account for heteroskedasticity of unknown forms, including spatial correlation of the error terms. Adjusted R^2^ values, while not identical in calculation to OLS R^2^ values, serve as a goodness-of-fit measure.

Table 2b gives the total effects of *TDN_W2_* and *TDN_W3_* on *HRY*. In this situation, total effects are defined as the sum of indirect and direct high temperature effects on *HRY*. The indirect effect refers to the change in chalk content multiplied by the effect of a one-unit change in chalk content on *HRY*. Direct effects are the effects of one-unit changes in *TDN_W2_* or *TDN_W3_* on *HRY*, holding constant the effect of the change in chalk content. The total effects of a one-unit increase in these variables on *MRY* are the direct temperature coefficients in the *MRY* model (Table 2c) because chalk is not included as an explanatory variable.

Table S2 in File S1 presents milling quality system estimates including the disaggregated *TD* and *TN* metrics of exposure to high temperature. Including the disaggregated metrics leads to nonsensical, statistically significant coefficients for all three milling quality outcomes. Unexpected, nonsensical coefficients in Table S2 are those that are statistically significant and negative. This means that more time spent at temperatures above the critical threshold (33°C day/22°C night) decreases chalk content, thus increasing milling quality, ceteris paribus. For *TD_W2_*, this occurs in Wells, LaGrue, and Jupiter (P<0.05) (Table S2 in File S1). This estimated reduction in *CHK* given higher daytime temperatures above the critical threshold at the early grain filling stage, is physiologically not possible as high temperatures during early grain filling have the largest impact on white core chalk formation due to increased assimilate demand and a shortened window of assimilate supply and deposition into the grains [15]. The combination of multicollinearity and over-fitting appears to cause these nonsensical estimates. The nonsensical estimates in the full model and the relatively small difference in goodness-of-fit between the full and *TN* specifications compared with the full and *TD* specification might lead one to drop the day-temperature variables and proceed with only night variables [11], [17]; however, this precludes any analysis of increased day temperatures. We aggregate *TD* and *TN* to allow changes in day and night temperatures to affect our outcome variables. Prediction based only on *TN* would capture shifts in only the night portions of the temperature curve.

### Combining Paddy Yield and Milling Quality

**Figure 3:**
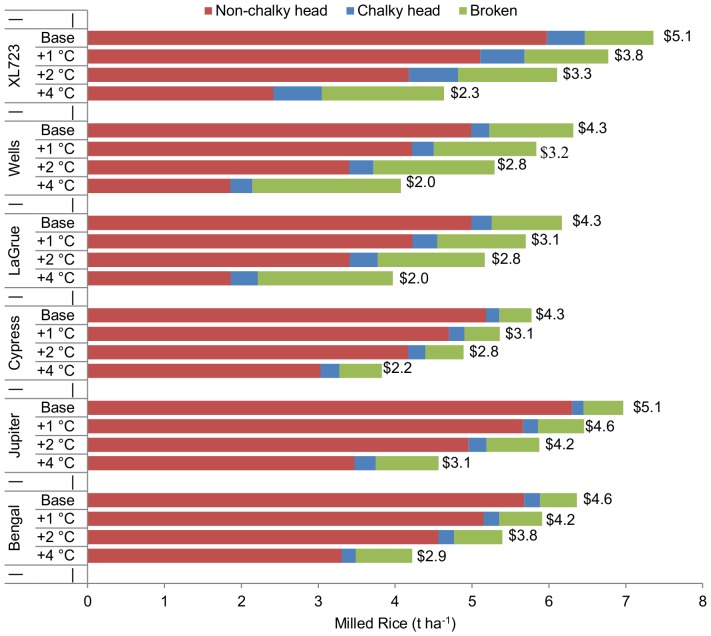
Nonlinear response of mean milled rice outcomes of tested genotypes given 1°C, 2°C, and 4°C increases in mean growing-season temperature. Average milled non-chalky head rice (red), chalky head rice (blue), and broken rice (green) per hectare were derived from estimated changes in paddy and milling quality outcomes, by scenario, using sample mean values of paddy and milling quality outcomes as baselines. Estimated changes in mean paddy and milling quality outcomes illustrated in Figures 2 and 3 were used to calculate the milled mass per hectare values illustrated above. Estimated changes in paddy yield from the baseline were calculated using the pooled sample of paddy yield trials and do not vary across cultivar. These changes are presented in Figure 2. Total milling revenue ($1,000 ha^−1^) is presented at the end of each bar. We used the sample means of milled medium-grain (Bengal and Jupiter) and long-grain (XL723, Wells, LaGrue, and Cypress) U.S. rice prices from 2007–2010, the sample period, for the price of non-chalky head rice, and the sample mean of Arkansas brewers’ milled rice prices over the 2007–2010 period for the price of broken and chalky-head rice kernels.

*CHK*, *MRY* and *HRY* largely determine the value of a unit of paddy rice, but the quantity of milled rice output per unit area harvested depends on paddy yield. Figure 3 illustrates XL723’s susceptibility to chalky kernel formation while maintaining high head rice potential per hectare due to its high yield. Cypress, the relatively low-yielding, high quality long-grain cultivar compares very well in non-chalky head rice production despite having a baseline yield disadvantage. The baseline estimate of XL723’s non-chalky milled head rice is higher than Cypress’s because of XL723’s 31% baseline paddy yield advantage over Cypress. The relative proportions of non-chalky and chalky head rice, and broken rice change as average growing-season temperature increases from the baseline level by 1°C, 2°C, and 4°C. Given a 4°C increase, Cypress is estimated to produce over 0.6 t ha^−1^ more non-chalky head rice than XL723 and roughly 1.2 t ha^−1^ more than LaGrue or Wells.

**Figure 4:**
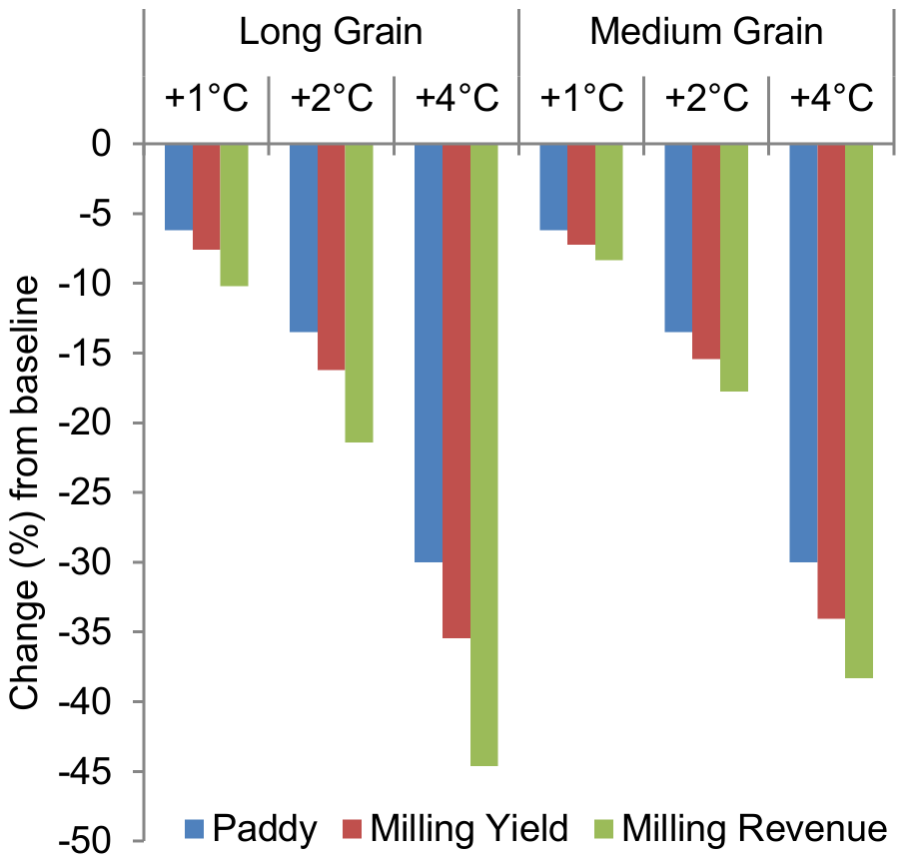
Changes in paddy and milling yield, and milling revenue across 1°C, 2°C, and 4°C increases in average growing-season temperature. The consequences of stopping the analysis at the paddy yield level are depicted above. Milling revenue lossses are greater for long-grain cultivars than medium-grain cultivars because high-temperature exposure causes much larger declines in *HRY* among long-grain cultivars. Medium grains are also less susceptible to chalk formation under heat-stressed conditions, but this disparity contributes relatively less to the disproportional response of milling revenue.

Total milling revenues across cultivars do not follow the same pattern as milled rice quantity because the quantities of broken and chalky kernels are valued less than the quantities of non-chalky kernels. Using the average U.S. FOB price for long- and medium-grain milled head rice and the average price for broken rice over the sample period, the total revenue estimates in Figure 3 illustrate the widening gap as hotter temperatures lead to quality reductions which further devalue the reduced quantity of paddy rice. Wells and XL723 illustrate this dynamic among long grain genotypes with 1°C and 2°C increases in average growing season temperature reducing paddy yield by 6.2% and 13.5%. Total milling output per hectare given these temperature increases declines by 7.6% and 16.2% in Wells and by 8% and 17% in XL723, respectively. These total quantity decreases do not illustrate the result that Wells is more susceptible to breaking during milling than XL723 (Fig. 3). The percentage changes in revenue per hectare, however, illustrate this point because we have discounted the price of broken and chalky kernels according to observed market outcomes. Cypress’s high milling yield and quality results in a relatively large percentage of revenue being derived from non-chalky head rice, but the paddy yield disadvantage leads to lower total revenue in all but the +4°C scenario, wherein the hybrid (XL723), in spite of a paddy yield advantage, records lower total revenue than Cypress. The percentage changes in paddy and milled rice yield and milling revenue presented in Fig. 4 illustrate the benefits of estimating paddy yield and milling quality responses to high-temperature stress. Across medium- and long-grain cultivars, the results show that paddy yield changes due to high-temperature stress understate the actual implications of a hotter growing season. For long- and medium-grain varieties, increasing average growing-season temperature by 1°C decreases milling revenue on average by 10% and 8%, respectively (Fig. 4). The small proportions of chalky and broken kernels in medium-grain relative to long-grain varieties (Fig. 3) cause these results.

## Discussion

### Paddy Yield

The statistically insignificant effect of high temperatures during the vegetative stage differs from [10], which found the most detrimental effect of high temperatures at the vegetative, but agrees in direction with [10]. We attribute this discrepancy to the ability of our *TDN* metric to capture heat effects on sensitive processes occurring during the reproductive and ripening stage more than do average daily temperatures. The statistical significance of the *TDN_W1_* coefficient supports this conclusion. The statistically significant *TDN_W1_* coefficient agrees with the experimental literature on the inverse relationship between high temperatures at pollen development and anthesis leading to increased spikelet sterility and lower yields due to loss of pollen viability [5], [26], poor anther dehiscence [31], pistil hyperplasia [32], pollen germination and pollen tube growth [26], [33] and/or abnormal fertilization [34]. High temperatures enhance senescence [35], by shortening active grain-filling duration and ultimately reducing grain weight and yield [36], [37], as shown by a negative, albeit statistically insignificant (P>0.10), *TDN_W2_* coefficient.

The estimated 6.2% decline in yield given a 1°C increase in growing season temperature splits the difference between the often-cited 10% decline found in [11] and the revised estimated decline of ∼5% put forth in [28] with data from [11]. We attribute the nonlinearity of yield response to 2°C and 4°C increases in growing-season temperature to our *TDN* metric, which captures the nonlinear changes in high-temperature accumulation (both frequency and magnitude) given shifts in the temperature curve (illustrated in Fig. S1), compared to just frequency when average temperatures are considered. That is the area between the critical threshold temperature (33°C/22°C) and the temperature curve increases nonlinearly as the average temperature increases linearly. As a result, our metric is nonlinear as a result of variable definition rather than parameterization of the yield-response models.

### Milling Quality

Among the different categories of chalk - white cored or milky white chalk determines kernel susceptibility to breakage and is known to increase significantly with heat stress during the early grain-filling stage (W2) [38], [39] caused primarily by loose amyloplast packing and not because of starch degradation by α-amylase [40]. Source-sink manipulation studies have established a close relationship between assimilate supply and white core chalk formation [39] with increasing assimilate supply overcoming chalk formation even under high temperatures [36]. Moreover, enhanced respiration under high temperatures alone could not account for assimilate losses [11], [37], [41]. White core chalk does not spread to the lateral parts of a grain even under high temperatures since the gaps between amyloplasts are much smaller than the larger amyloplast at the center, thereby reducing chalk formation in the lateral sections [40]. Increased white core chalk formation at W2 leads to an increased reduction in *HRY* and *MRY* while high temperature corresponding to the formation of white back or basal white chalky kernels (W3) did not result in an equivalent reduction in *HRY* and *MRY* (Table 2b,c), since chalk formed during W3 does not directly contribute to kernel breakage [39], but reduces the market value of head rice. Hence the type and the location of chalk formation are crucial and this determines the degree of kernel breakage while milling.

Using a predictive model of chalk content at the prefecture level in Japan, [42] reconfirmed that the rice plant is most sensitive to high temperature induced chalk content formation during the 30 days immediately following heading. Our finding that W2 is the period during which high temperatures have the greatest impact on white core chalk content leading to increased milling losses agrees with [42]. However, the narrow focus on milling quality in [42] disregards the importance of paddy yield. Future research on climate change’s effects on rice production might benefit from the application of our methodology to combined paddy and milling output data at a larger spatial scale, augmenting our approach with the elements of [42] that improve prediction on a regional scale.

### Paddy Yield and Milling Quality

Combining paddy yield and milling quality data offers our greatest contribution to the literature. Rice is a unique cereal consumed primarily as intact kernels and hence post-milling kernel appearance and dimensions primarily determine the market price and producer revenue. Decreases in milling quality attributable to high temperature exacerbate the more commonly modeled detrimental impacts of high-temperature stress solely on yield [10], [11]. Changes in the distribution of milled rice among non-chalky and chalky *HRY* and broken rice tell only part of the story. The total revenue implications associated with changes in this distribution drive the wedge further between high-temperature exposure in the paddy and milled outcome levels with the size of this wedge varying across genotypes. We illustrate this using multiple regression models developed above to estimate the impacts of 1°C, 2°C, and 4°C increases in growing season temperatures on the changes in quantity, quality distribution, and total revenue at the milled level (Fig. 3). We choose static prices because the effect of climate change on prices depends on global grain supply and demand outcomes. A review of the previous literature on such outcomes has shown the associated price changes to be either small in magnitude and/or conflicting in direction [43].

Climate change necessitates the development of cultivar-specific models capable of simultaneously estimating the relationship between high temperatures and rice yield and milling quality. The omission of rough rice yield or milling quality inevitably leads to an underestimation of the true extent of high-temperature effects on rice production and this could distort policy decisions designed to overcome food insecurity given the changing climate. Biologically, the estimation of both yield and milling quality responses to high temperatures is critical because the detrimental impacts occur across various developmental stages and are genotype-specific. High-temperature stress during the sensitive gametogenesis and early flowering largely determines paddy yield loss while increased chalk formation (white core) during the early grain-filling stage governs head rice loss due to breakage. Genotypic variation illustrated in our milling quality results might provide rice breeders with insight to justify genetic crosses of lower yielding varieties (e.g. Cypress) if the goal is maximization of non-chalky *HRY* or to select high-yielding hybrid varieties (e.g. XL723) to maximize edible rice (calories) per unit land area. As mentioned earlier, impact analysis of high temperature under field conditions across the major rice-producing regions of South and Southeast Asia has been restricted to grain yield [10], [11], which are in agreement with the degree of decline presented in our study, using similar thresholds. Hence, the impact on the overall milling outcomes and economic losses could be approximated, which would increase the negative impact of higher temperatures on global food security since rice is the world’s most important staple cereal.

This study provides insight where previous experimental and econometric analyses of rice production outcomes have not: (i) examined, or if they have examined, have not estimated, rough rice yield and milling quality models simultaneously; (ii) extensively addressed multicollinearity issues among explanatory weather variables in econometric models; and (iii) identified the economic implications of these innovations. Continued observation of the interaction between increasingly variable weather conditions and rice production outcomes will allow refinement and enhancement of this modeling approach to, we hope, provide plant breeders, agricultural policy makers, and private enterprises with important direction for sustaining rice production in an increasingly hot future.

## Supporting Information

Figure S1 **Visual approximation of exposure to high temperatures.** The black line represents temperatures at time *t,* the upper (lower) horizontal line represent the day (night) temperature threshold, and the red (orange) area between the temperature and upper (lower) horizontal line represents the day (night) high-temperature exposure, expressed in this study as *TD­_33_* (*TN_22_*). The summation of these areas gives the *TDN* metric used in this paper’s primary analysis. Artificially increasing growing-season temperatures, thus shifting the temperature curve upward, allows examination of non-linear responses to high-temperature exposure.
(PDF)

File S1 Tables S1. Coefficients from primary specification regression. Table S1 contains the coefficients used to generate Figure 2. Table S2.Coefficients from alternate specification regression. Table S2 contains coefficients from the alternate specification of the milling quality model.
(DOCX)
