# Supplementary figures and images for: Neglecting Rice Milling Yield and Quality Underestimates Economic Losses from High-Temperature Stress
Source: PLoS One. 2013 Aug 22;8(8):e72157. doi: 10.1371/journal.pone.0072157 (PMC3750041; doi:10.1371/journal.pone.0072157)

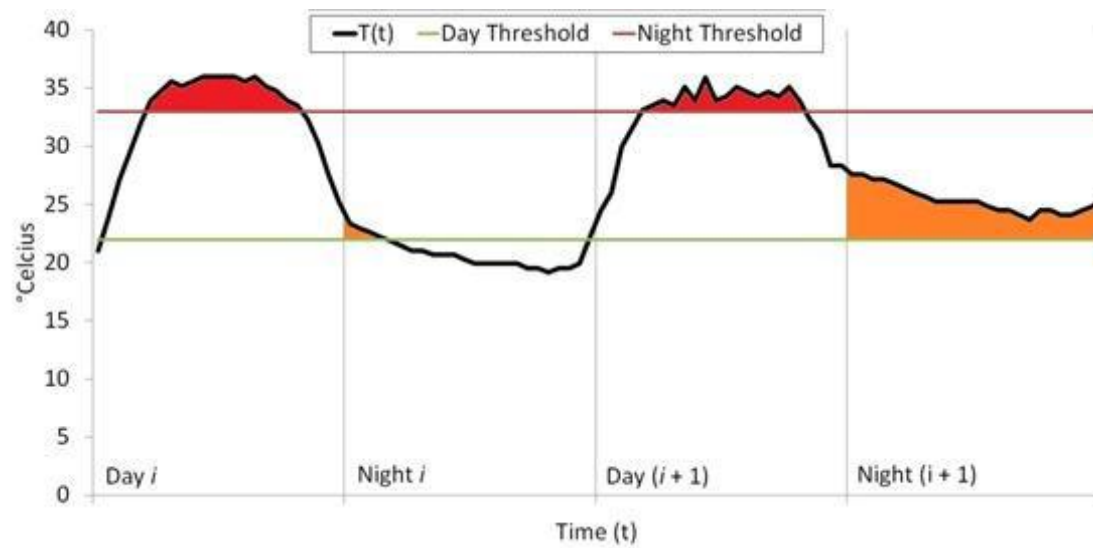

Figure S1. Visual approximation of exposure to high temperatures

Supplement: Figure S1 — Visual approximation of exposure to high temperatures. The black line represents temperatures at time t, the upper (lower) horizontal line represent the day (night) temperature threshold, and the red (orange) area between the temperature and upper (lower) horizontal line represents the day (night) high-temperature exposure, expressed in this study as TD­33 (TN22). The summation of these areas gives the TDN metric used in this paper’s primary analysis. Artificially increasing growing-season temperatures, thus shifting the temperature curve upward, allows examination of non-linear responses to high-temperature exposure. (PDF) [file pone.0072157.s001.pdf]
