# Supplementary material for: Neglecting Rice Milling Yield and Quality Underestimates Economic Losses from High-Temperature Stress
Source: PLoS One. 2013 Aug 22;8(8):e72157. doi: 10.1371/journal.pone.0072157 (PMC3750041; doi:10.1371/journal.pone.0072157)
Supplement: File S1 — Tables S1. Coefficients from primary specification regression. Table S1 contains the coefficients used to generate Figure 2. Table S2.Coefficients from alternate specification regression. Table S2 contains coefficients from the alternate specification of the milling quality model. (DOCX) [file pone.0072157.s002.docx]

Table S1. Primary and alternate specfications of the paddy yield model

|  | Model Specification | | |
| --- | --- | --- | --- |
| *Z* Variable | (1)^a^ | (2) | (3) |
| *$\bar{T}_{V}$* | -0.007 | -0.048*** | -0.011 |
| *$\overline{SR}_{V}$* | 0.027** | -0.017*** | 0.002 |
| *TDN_W1_* | -0.003*** | --- | -0.003*** |
| *TDN_W2_* | -0.001 | --- | -0.001 |
| *TDN_W3_* | 0.001 | --- | 0.000 |
| *VPD_W1_* | --- | 0.092 | 0.315** |
| *VPD_W2_* | --- | -0.068 | -0.262 |
| *VPD_W3_* | --- | 0.278*** | 0.132 |
| *$\overline{SR}_{W4}$* | 0.037*** | -0.014 | 0.038** |
| *ln*(*HMC*) | -0.128*** | -0.18*** | -0.132** |
| *XL723* | 0.136*** | 0.159*** | 0.141*** |
| *Jupiter* | 0.089*** | 0.099*** | 0.091*** |
| *Bengal* | 0.005 | 0.018 | 0.008 |
| *Wells* | 0.015 | 0.008 | 0.015 |
| *COR* | 0.162*** | 0.165*** | 0.107** |
| *KSR* | 0.285*** | 0.236*** | 0.251*** |
| *NPT* | 0.324*** | 0.238*** | 0.253*** |
| *PT* | 0.128*** | 0.112*** | 0.118*** |
| *STGT* | 0.183*** | 0.138*** | 0.162*** |
| Intercept | 8.167*** | 11.125*** | 8.771*** |
| Adjusted R^2^ | 0.642 | 0.629 | 0.645 |
| F-statistic | 33.2 | 31.4 | 28.4 |
| N | 288 | 288 | 288 |

Ordinary least squares (OLS) regression results from the estimation of the (a) primary (1) and alternate (2 and 3) specifications of the paddy yield model. Specification (2) includes mean vapor pressure deficit (*VPD*), a function of temperature and relative humidity during W1, W2, and W3, denoted by subscript. Specification (3) includes both mean vapor pressure deficit and the thermal exposure measure used in the primary model. LaGrue and Rohwer, AR, USA, were used as the base groups for the cultivar and station fixed-effects, respectively. *,**, and *** denote statistical significance at the 0.10, 0.05, and 0.01 levels, respectively. Heteroskedasticity robust standard errors were used to calculate the t-ratios for tests of statistical significance.

Table S2. Milling quality system estimated including TD and TN as explanatory variables

| **a.** ***CHK*** | Bengal | Jupiter | Cypress | LaGrue | Wells | XL723 |
| --- | --- | --- | --- | --- | --- | --- |
| *Intercept* | -1.74 | -14.9*** | -10.8*** | -30.1*** | -20.0*** | -44.3*** |
| *TD_W2_* | -0.037 | -0.532*** | 0.107 | -0.599** | -0.703*** | 0.068 |
| *TD_W3_* | 0.186** | 0.423*** | -0.179 | 0.606** | 0.462*** | 0.436* |
| *TN_W2_* | 0.092 | 0.746*** | 0.362*** | 1.33*** | 1.08*** | 0.679*** |
| *TN_W3_* | -0.012 | 0.203 | 0.415*** | 0.338 | 0.399** | 1.03*** |
| *COR* | -0.732*** | -0.157 | -1.04*** | -0.208 | -1.57*** | -0.585 |
| *KSR* | 0.457 | 0.660** | -0.963** | 1.052 | 2.16** | 4.33*** |
| *NPT* | -1.87*** | -1.50** | -1.18** | -4.22*** | -2.99*** | -1.99*** |
| *PT* | -0.478* | 0.542* | -1.19*** | -1.15* | -0.877** | 2.03*** |
| *STGT* | -0.877*** | -1.19*** | -1.78*** | -3.16*** | -2.36*** | -0.800 |
| Adj. R^2^ | 0.406 | 0.747 | 0.613 | 0.751 | 0.660 | 0.774 |
| **b.** ***HRY*** | Bengal | Jupiter | Cypress | LaGrue | Wells | XL723 |
| *Intercept* | 80.5*** | 70.7*** | 87.7*** | 110.5*** | 51.4*** | 117.7*** |
| *TD_W2_* | 0.871*** | 0.700 | 1.723*** | 1.129** | 0.692* | -0.351 |
| *TD_W3_* | 0.016 | -0.032 | 0.129 | 0.651 | 0.229 | 0.571 |
| *TN_W2_* | -2.48*** | -1.59*** | -3.585*** | -3.89*** | -2.46*** | -1.75*** |
| *TN_W3_* | -0.238 | 0.007 | -0.885** | -0.87** | -0.218 | -1.52*** |
| *CHK* | -0.495 | -1.23*** | 0.601* | -0.791*** | -1.65*** | -0.375** |
| *HMC* | 2.09** | 1.44** | 2.079** | 1.05 | 4.79*** | 1.65* |
| *HMC^2^* | -0.046** | -0.034** | -0.05** | -0.031 | -0.112*** | -0.046* |
| *COR* | 1.409 | 0.511 | 3.597*** | -3.62* | -2.64** | -0.792 |
| *KSR* | -6.25** | -7.49*** | -7.495** | -9.99*** | -8.53*** | -9.01*** |
| *NPT* | 0.146 | -1.596 | 0.772 | -3.62 | -2.62 | -0.916 |
| *PT* | 0.721 | 0.914 | 1.553* | -0.617 | 0.617 | 0.259 |
| *STGT* | 1.73** | 0.112 | 2.14** | 2.40** | 2.89*** | 1.05 |
| Adj. R^2^ | 0.500 | 0.463 | 0.623 | 0.827 | 0.801 | 0.711 |
| **c.** ***MRY*** | Bengal | Jupiter | Cypress | LaGrue | Wells | XL723 |
| *Intercept* | 88.6*** | 94.0*** | 86.0*** | 93.8*** | 94.59*** | 104.3*** |
| *TD_W2_* | 0.287 | 0.408** | 0.763*** | 0.384*** | 0.395*** | 0.037 |
| *TD_W3_* | -0.029 | -0.075 | -0.262 | -0.165 | 0.015 | 0.435*** |
| *TN_W2_* | -0.925*** | -1.35*** | -1.21*** | -1.17*** | -1.06*** | -1.12*** |
| *TN_W3_* | -0.042 | 0.153 | -0.102 | -0.093 | -0.396*** | -0.851*** |
| *HMC* | -0.131*** | -0.283*** | -0.067* | -0.197*** | -0.169*** | -0.211*** |
| *COR* | 1.57*** | 1.34*** | 1.69*** | 1.55*** | 1.76*** | 1.48*** |
| *KSR* | -3.73** | -5.12*** | -4.70*** | -2.88*** | -4.33*** | -3.13* |
| *NPT* | 0.365 | 0.203 | 1.116 | 0.586 | 0.183 | 0.585 |
| *PT* | 0.577* | 1.036** | 0.82*** | 0.598* | 1.089*** | 0.514 |
| *STGT* | 0.536* | 1.361*** | -0.175 | 0.531 | 0.791** | 1.03*** |
| Adj. R^2^ | 0.626 | 0.760 | 0.697 | 0.749 | 0.678 | 0.689 |
| N | 118 | 112 | 117 | 100 | 137 | 125 |

Marginal effect coefficients can be interpreted as the percentage point change in the dependent variable (bolded) given a one-unit change in the explanatory variable. The marginal effects of *CHK* and *HMC* on *HRY* are given in **b** and *HMC* on MRY are given in **c**. Station fixed-effect estimates have been omitted from this table. We estimated the system using Generalized Method of Moments (GMM) estimation to account for heteroskedasticity of unknown forms, including spatial correlation of the error terms. *,**, and *** denote statistical significance at the 0.10, 0.05, and 0.01 levels, respectively. Heteroskedasticity robust standard errors were used to calculate the t-ratios for tests of statistical significance. Adjusted R^2^ values, while not identical in calculation to OLS adjusted-R^2^ values, serve as a goodness-of-fit measure.
